# Assessment of the diagnostic value of serum ceruloplasmin for Wilson’s disease in children

**DOI:** 10.1186/s12876-022-02186-0

**Published:** 2022-03-16

**Authors:** Xinshuo Lu, Simin Li, Wen Zhang, Yunting Lin, Zhikun Lu, Yanna Cai, Xueying Su, Yongxian Shao, Zongcai Liu, Huiying Sheng, Yonglan Huang, Li Liu, Chunhua Zeng

**Affiliations:** Department of Genetics and Endocrinology, Guangzhou Women and Children’s Medical Center, Guangzhou Medical University, 9 Jinsui Rd, Guangzhou, 510623 China

**Keywords:** Ceruloplasmin, Wilson’s disease, Children, Diagnosis

## Abstract

**Background:**

Serum ceruloplasmin is one of the major diagnostic parameters for Wilson’s disease (WD). Age and gender difference of serum ceruloplasmin remain controversy. This study aims to assess diagnostic value of serum ceruloplasmin level for WD in children up to age of 15 years.

**Methods:**

Serum ceruloplasmin levels were measured in 317 WD patients, 21 heterozygotes, 372 healthy control children and 154 non-WD patients with other liver diseases. Receiver operating characteristic (ROC) curve was used to determine the diagnostic accuracy of serum ceruloplasmin for WD in children.

**Results:**

Among healthy controls, serum ceruloplasmin level was slightly low in the infants younger than 6 months, and then maintained from 26 to 33 mg/dl after age of 6 months. A total of 8.1% of healthy children had levels of serum ceruloplasmin < 20 mg/dL. Serum ceruloplasmin level was 5.7 ± 4.7 mg/dl in WD patients, and 25.6 ± 5.9 mg/dl in heterozygous carriers. Only 1.9% of WD patients had serum ceruloplasmin levels > 20 mg/dL. Serum ceruloplasmin levels had gender difference, being higher in healthy boys than healthy girls, and higher in asymptomatic WD boys than asymptomatic WD girls (*p* < 0.01, *p* < 0.05). Serum ceruloplasmin levels also presented genotypic difference. WD patients with R778L homozygotes exhibited lower levels of serum ceruloplasmin than the patients without R778L (*p* < 0.05). The ROC curve revealed that serum ceruloplasmin level, at a cutoff value of 16.8 mg/dL, had the highest AUC value (0.990) with a sensitivity of 95.9% and a specificity of 93.6%.

**Conclusions:**

Serum ceruloplasmin is one of sensitive diagnostic biomarkers for WD in children. Gender and genotypic difference of serum ceruloplasmin level should be considered. The cutoff value of serum ceruloplasmin level < 16.8 mg/dL may provide the highest accuracy for diagnosis of WD in children.

**Supplementary Information:**

The online version contains supplementary material available at 10.1186/s12876-022-02186-0.

## Introduction

Wilson’s disease (WD) is an autosomal recessive disorder of copper metabolism caused by mutations in the *ATP7B* gene [1]. The ATP7B protein is a copper-transporting ATPase expressed predominantly in the liver and to a lesser extent in most other tissues. Mutations in the ATP7B gene lead to failure of copper transport from hepatocytes into bile, resulting in Wilson disease, a copper toxicity disorder characterized by a dramatic build-up of intracellular hepatic copper with subsequent hepatic and neurological abnormalities. If WD is not recognized and treated early, hepatic and neurologic damage may be rapid and fatal. Therefore, early diagnosis is crucial to prevent the progression of the disease and to approach a good outcome of WD.

Ferenci et al*.* proposed a conventional diagnostic scoring system for WD, including low serum ceruloplasmin levels, elevated urinary copper excretion, KF ring, liver copper content and genetic findings [2]. Socha et al. further published a position paper of specific diagnostic criteria for WD in children [3]. With the application of Ferenci scoring system, WD can be identified and diagnosed easily when the patients present with typical symptoms. However, the diagnosis of WD is not easy in asymptomatic children who presented with no clinical manifestation but isolated elevation of serum aminotransferases, and in patients in the situation of acute liver failure [4–6].

Ceruloplasmin is the main copper-carrying protein in blood. With the defect of copper transportation, non-copper bound apo-ceruloplasmin is rapidly degraded [7]. Serum ceruloplasmin level is typically decreased in most of WD patients, but not in all of them. On the other hand, serum ceruloplasmin level may vary with age, change with inflammation or estrogen, and decrease in some *ATP7B* heterozygous carriers or in other conditions with liver or renal disease [8, 9]. According to the diagnostic consensus, serum ceruloplasmin level < 20 mg/dL is conventionally considered as one of the major diagnostic thresholds for WD [2]. Socha et al. suggested that WD should be highly suspected with serum ceruloplasmin level < 10 mg/dl in children [7]. Although serum ceruloplasmin is never alone criteria for the diagnosis of WD, it is one of the major diagnostic parameters for reflecting abnormal copper metabolism. There have been few studies evaluating the diagnostic accuracy of serum ceruloplasmin in WD children [10–14]. Therefore, it is important to establish the age and gender specific reference of serum ceruloplasmin levels in healthy children, and evaluate the diagnostic criteria of serum ceruloplasmin level for WD during early childhood.

In our previous study, we have noticed that serum ceruloplasmin level is decreased in the majority of WD children [15]. This study aims to provide reference value of serum ceruloplasmin level in healthy children stratified by age and gender, and assess diagnostic criteria of serum ceruloplasmin level for screening and early diagnosis of WD in children.

## Materials and methods

### Study subjects

A total of 317 children with WD diagnosed at Guangzhou Women and Children’s Medical Center (GWCMC) from January 1, 2010 to December 31, 2020 were enrolled in this study (n = 317). The diagnosis of all 317 WD patients were established on a total of score of four or more points according to the consensus for WD [2, 7]. These patients were categorized into three groups based on clinical manifestations at diagnosis: (1) Asymptomatic WD, including 236 of whom presented with isolated elevation of serum aminotransferases through occasional tests and 25 diagnosed through family screening (n = 261); (2) WD with acute liver failure (n = 15); (3) other types of WD, including acute hepatitis, chronic hepatic disease, neurological disease, renal disease and purpura (n = 41).

Heterozygous group (n = 21) included unaffected siblings carrying one pathogenic mutation in the *ATP7B* gene. Non-WD patients were represented by pediatric patients of GWCMC with exclusion of WD, including acute liver failure by infection or acetaminophen (n = 20), viral hepatitis (n = 38) and nephrotic syndrome (n = 96). Data of the patients with non-WD diseases were retrieved from database of clinical lab in GWCMC.

Healthy controls (n = 372) included 360 children who came for routine physical examination and 12 wild-type siblings of WD patients. Blood samples in healthy controls were originally collected for liver function test and then used for further measurement of serum ceruloplasmin. This study was approved by the ethical clinical research committee of Guangzhou Women and Children’s Medical Center, and appropriate informed consents were obtained parental permission for all participants.

### Biochemical and genetic analysis

The clinical variables of WD patients and their siblings, including age, sex, family history, clinical manifestations, and physical examinations, were collected from participant medical history and chart record at diagnosis. Serum ceruloplasmin levels were measured by immunonephelometry using the Beckman Coulter Immage 800 with standard reagents (Beckman Coulter, Brea, CA, USA).

Genetic analyses for the *ATP7B* gene mutation were performed in all WD patients and their siblings. Genomic DNA was extracted from peripheral blood samples. DNA sequencing was applied to the entire coding regions of exons 1–21 of the ATP7B gene with adjoining intron boundaries. The exported sequences were aligned and inspected with the reference sequence using DNAMAN software.

### Statistical analysis

Statistical analyses were performed using SPSS (version 25; SPSS Inc., Chicago, IL, USA) and GraphPad Prism (version 7.0; GraphPad Software, La Jolla, California, USA). Continuous quantitative variables (age, serum Ceruloplasmin) were expressed as the means and standard deviations (SD). Sensitivity, specificity, positive predictive value (PPV) and negative predictive value (NPV) were calculated for assessment of diagnostic accuracy. The receiver operating characteristic (ROC) curve and the corresponding area under the curve (AUC) were used to determine the optimal cutoff value of serum ceruloplasmin for diagnosis of WD. A value of *p* < 0.05 was considered statistically significant.

## Results

### Serum ceruloplasmin in healthy children

Serum ceruloplasmin levels were measured in 372 healthy children from the age of newborn to 15 years. Of these participants, 218 (58.6%) were boys, with a mean age of 6.8 ± 4.2 years and 154 (41.4%) were girls, with a mean age of 6.2 ± 3.8 years. The mean level of serum ceruloplasmin in these healthy controls was 30.7 ± 7.8 mg/dL. Among them, 30(8.1%) had serum ceruloplasmin level < 20 mg/dL, whereas 13 (3.5%) had the level < 15 mg/dL.

**Fig. 1:**
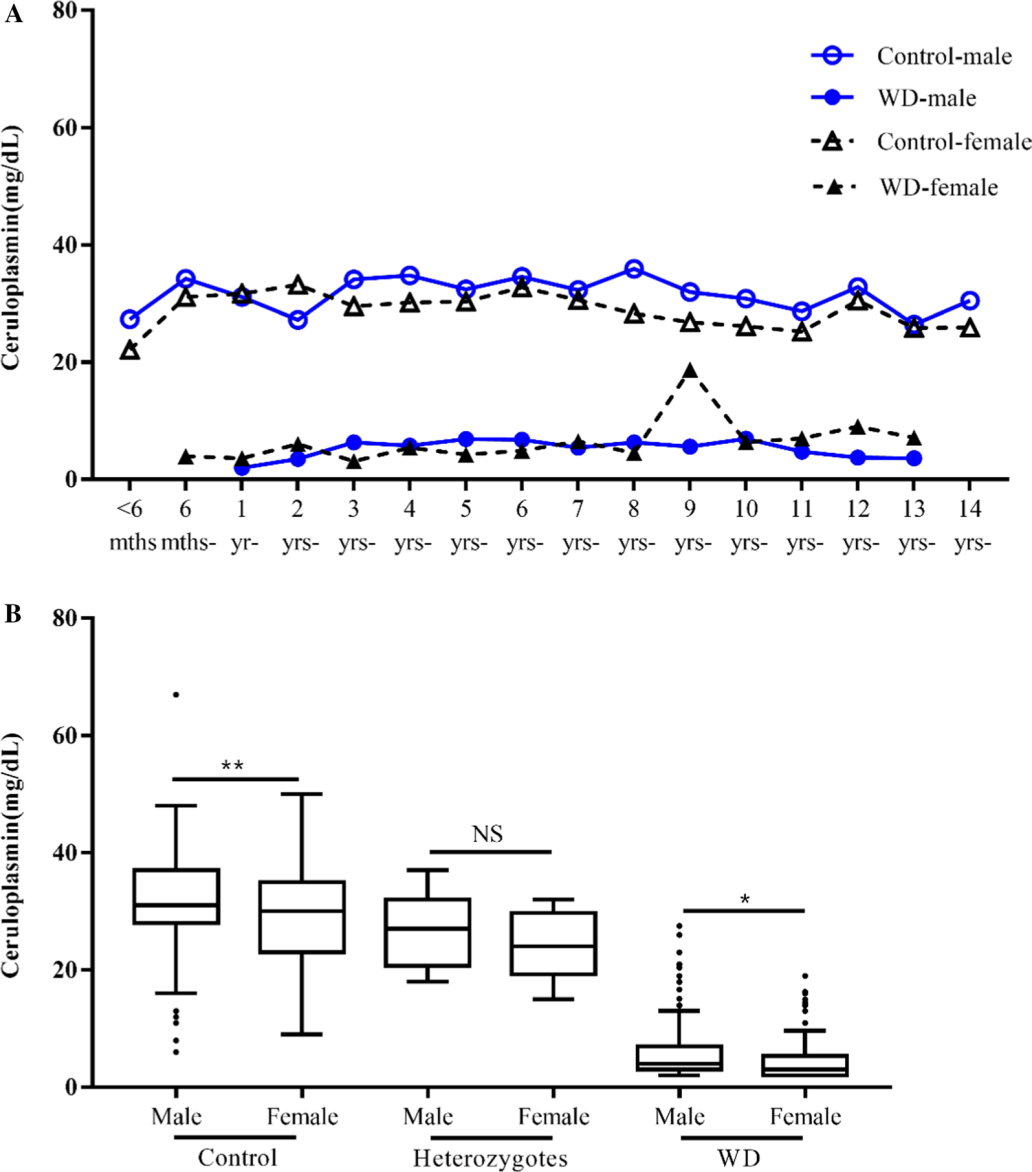
Gender difference of serum ceruloplasmin was analyzed among healthy children, WD patients and heterozygotes. **A** There were no significant gender differences of serum ceruloplasmin levels intra each age subgroup among WD patients and among healthy children. **B** Serum ceruloplasmin level was higher in boys than girls among healthy children (*p* < 0.01) and also among WD patients (*p* < 0.05). The mean levels of serum ceruloplasmin in WD patients were much lower than that in healthy controls and heterozygotes

Age specific reference value of serum ceruloplasmin level was analyzed (Table 1 and Fig. 1). These healthy children were partitioned into 16 age subgroups. Serum ceruloplasmin was at the lowest level (25.5 ± 10.1 mg/dL) in children younger than 6 months, then rapidly increased to 33.0 ± 9.3 mg/dL by the age of 1 year, and maintained from 26 to 33 mg/dl after age of 6 months. There was no age specific difference of serum ceruloplasmin levels in healthy children (*p* > 0.05).

**Table 1:** Serum ceruloplasmin levels in healthy children and WD patients stratified by age

Age group	Healthy children							WD patients							*p* value
	Cp(mg/dL)	Number	0–5	5–10	10–15	15–20	≥ 20	Cp(mg/dL)	Number	0–5	5–10	10–15	15–10	≥ 20	
< 6 mths	25.5 ± 10.1	20	0	1	3	2	14	–	–	–	–	–	–	–	–
6 mths-	33.0 ± 9.3	20	0	0	1	2	17	4.0	1	1	0	0	0	0	–
1 yr-	31.5 ± 8.0	28	0	0	0	3	25	3.3 ± 2.2	4	3	1	0	0	0	*p* < 0.001
2 yrs-	30.0 ± 8.3	22	0	1	1	0	20	4.6 ± 3.3	19	13	5	0	1	0	*p* < 0.001
3 yrs-	32.0 ± 6.4	24	0	0	0	1	23	5.2 ± 5.1	51	33	12	3	0	3	*p* < 0.001
4 yrs-	33.7 ± 6.5	25	0	0	0	0	25	5.6 ± 4.4	59	35	15	5	3	1	*p* < 0.001
5 yrs-	31.7 ± 5.7	35	0	0	0	1	34	6.5 ± 5.6	48	25	12	8	1	2	*p* < 0.001
6 yrs-	33.7 ± 5.0	26	0	0	0	0	26	6.3 ± 5.1	38	20	10	5	3	0	*p* < 0.001
7 yrs-	31.6 ± 5.3	25	0	0	0	0	25	5.3 ± 3.7	25	16	4	5	0	0	*p* < 0.001
8 yrs-	30.9 ± 5.9	27	0	0	0	1	26	6.2 ± 4.8	20	10	6	2	2	0	*p* < 0.001
9 yrs-	28.9 ± 6.3	22	0	0	0	2	20	7.6 ± 5.2	8	3	3	1	1	0	*p* < 0.001
10 yrs-	29.1 ± 9.8	21	0	0	3	2	16	6.5 ± 4.5	17	9	4	2	2	0	*p* < 0.001
11 yrs-	27.5 ± 7.7	23	0	1	1	0	21	6.3 ± 3.2	9	4	3	2	0	0	*p* < 0.001
12 yrs-	32.4 ± 8.8	25	0	0	0	0	25	4.9 ± 3.2	13	9	2	2	0	0	*p* < 0.001
13 yrs-	26.3 ± 6.2	16	0	0	0	1	15	6.4 ± 4.3	3	1	1	1	0	0	*p* < 0.001
14 yrs-	29.1 ± 9.7	13	0	0	1	2	10	3.0 ± 1.0	2	2	0	0	0	0	*p* = 0.003
Total	30.7 ± 7.8	372	0	3	10	17	342	5.7 ± 4.7	317	184	78	36	13	6	*p* < 0.001

Cp, ceruloplasmin; 0–5, 5–10, 10–15, 15–20 and ≥ 20 represented the ranges of serum ceruloplasmin (mg/dL); Number represented the number of the children with specific range of serum ceruloplasmin

Gender specific reference value of serum ceruloplasmin was also analyzed in these children (Table 1 and Fig. 1). The mean level of serum ceruloplasmin in healthy boys and girls were 31.8 ± 7.7 mg/dL and 29.2 ± 7.6 mg/dL respectively, exhibiting statistically significant gender difference (*p* < 0.01). While analyzing gender difference in each age subgroup, serum ceruloplasmin also exhibited higher level in boys than girls, but there was no significance with the exception of 8- year subgroup (*p* > 0.05).

### Serum ceruloplasmin levels in children with Wilson’s disease

As shown in Table 1, a total of 317 children were diagnosed as WD in GWCMC in last 11 years, 200 (63.1%) boys and 117 (36.9%) girls. The ratio of male to female in WD patients was 1.7:1. At diagnosis, the mean age of these patients was 6.1 ± 2.8 years (ranging 0.6 to 15.0 years). The mean level of serum ceruloplasmin in WD patients was 5.7 ± 4.7 mg/dL, which was significantly lower than that in healthy control subjects (*p* < 0.001). A total of 13 WD patients had serum ceruloplasmin between 15 and 20 mg/dL, 3 of whom presented with acute liver failure and 10 were asymptomatic (Additional file 1: Table S1). A total of 6 (1.9%) in 317 WD patients had normal value of serum ceruloplasmin > 20 mg/dL, all of whom were asymptomatic (6/261, 2.3%).

Age specific difference of serum ceruloplasmin level in WD patients was analyzed (Table 1 and Fig. 1). The youngest WD patient was a 7-month-old girl diagnosed through family screening. She was asymptomatic with low serum ceruloplasmin of 4 mg/dL but normal liver function. Among 317 WD patients, the mean level of serum ceruloplasmin varied in different age subgroups. No age specific difference of serum ceruloplasmin level was shown (*p* > 0.05).

Gender specific difference of serum ceruloplasmin level in WD patients was also analyzed. Of the 317 WD patients, the mean age of boys was 6.2 ± 2.8 years, and that of girls was 5.9 ± 2.9 years. There was no significant gender difference of serum ceruloplasmin among 317 WD patients and inside each age subgroup (Fig. 1). However, among 261 (82.3%) asymptomatic WD patients, serum ceruloplasmin level in 164 boys (6.0 ± 5.1 mg/dL) was significantly higher than that in 97 girls (4.6 ± 3.7 mg/dL) (*p* < 0.05), exhibiting similar trend with that in healthy children.

The association between clinical manifestation and the level of serum ceruloplasmin in WD patients was analyzed (Table 2). Of 261 asymptomatic WD children, serum ceruloplasmin was significantly lower and the age at diagnosis was much younger while compared with 56 WD children with various clinical symptoms and signs (*p* < 0.05, *p* < 0.001). Of 15 WD children with acute liver failure, serum ceruloplasmin was significantly higher and the age at diagnosis was much older while compared with 302 WD children without acute liver failure (*p* < 0.01, *p* < 0.001). None of WD patients with acute liver failure had serum ceruloplasmin level > 20 mg/dL.

**Table 2:** Serum ceruloplasmin levels in WD children with different manifestations at diagnosis

WD patients	Cp (mg/dL)	Age (years)	No. of patients					
			Total	0–5	5–10	10–15	15–20	≥ 20
Asymptomatic WD	5.5 ± 4.6^a^	5.4 ± 2.3^b^	261	157	67	22	9	6
Symptomatic WD	7.0 ± 4.8	9.4 ± 2.6	56	27	11	14	4	0
WD w/o ALF	5.6 ± 4.6^c^	6.0 ± 2.8^d^	302	180	75	30	11	6
WD w/ ALF	9.3 ± 4.7	9.0 ± 2.2	15	4	3	6	2	0
Total	5.7 ± 4.7	5.7 ± 4.7	317	184	78	36	13	6

WD, Wilson’s disease; ALF, acute liver failure

^a^Represents comparison of serum ceruloplasmin in asymptomatic WD with symptomatic WD children, *p* value < 0.05

^b^Represents comparison of age in asymptomatic WD with symptomatic WD children, *p* value < 0.001

^c^Represents comparison of serum ceruloplasmin between WD children with and without acute liver failure, *p* value < 0.01

^d^Represents comparison of age between WD children with and without acute liver failure, *p* value < 0.001

### Serum ceruloplasmin levels in non-WD patients with renal or hepatic diseases

Serum ceruloplasmin level was measured in 154 patients with non-WD diseases, including acute liver failure, viral hepatitis and nephrotic syndrome. The mean age of these non-WD patients was 4.9 ± 3.7 years (ranging from 1 month – 15.3 years) (Table 3). All non-WD patients had serum ceruloplasmin level > 20 mg/dL, with the mean level of serum ceruloplasmin (28.6 ± 11.8 mg/dL) much higher than that in WD patients (*p* < 0.001), but lower than healthy controls (*p* < 0.001).

**Table 3:** Serum ceruloplasmin levels in WD patients and non-WD diseases

Group	Cp (mg/dL)	No. of patients						*p* value
		Total	0–5	5–10	10–15	15–20	≥ 20	
Healthy children	30.7 ± 7.8	372	0	3	10	17	342	
WD patients	5.7 ± 4.7	317	184	78	36	13	6	*p < 0.001
Non-WD ALF	27.4 ± 12.7	20	0	0	3	3	14	*p > 0.05
Viral hepatitis	32.5 ± 7.9	38	0	0	1	2	35	*p > 0.05
Nephrotic syndrome	26.1 ± 9.0	96	0	0	7	13	76	*p < 0.001
Total	27.8 ± 9.7	154	0	0	11	18	125	

WD, Wilson’s disease; Cp, ceruloplasmin; ALF, acute liver failure

*p value while compared with healthy control

### Serum ceruloplasmin levels in WD patients with R778L in the *ATP7B* gene

Of the 317 WD patients, 313 had genetic testing for pathogenic mutations in the *ATP7B* gene, with 307 bi-allelic mutations and 6 one allelic mutation. Through family screening, 21 heterozygotes and 12 wild type siblings were identified. All wild type siblings were healthy and included in healthy controls. All heterozygotes were healthy without liver dysfunction. The mean level of serum ceruloplasmin in heterozygotes was (25.6 ± 5.9 mg/dL), significantly lower than that of healthy controls (30.7 ± 7.8 mg/dL, *p* < 0.05), but much higher than WD patients (5.7 ± 4.7 mg/dL, *p* < 0.001) (Fig. 1).

Among 313 WD patients, the most frequent mutation in the *ATP7B* gene was R778L, identified in 33.9% (106 patients) of 313 WD patients (Table 4). Of the 313 WD patients, 9 carried bi-allelic R778L mutations (R778L homozygotes), 97 had one allelic mutation of R778L together with one allelic mutation other than R778L (R778L heterozygotes) and 207 had none of R778L (No R778L). The patients with R778L homozygotes had the lowest level of serum ceruloplasmin (2.3 ± 0.5 mg/dL) and significantly lower than that of WD children without R778L (6.2 ± 4.8 mg/dL) (*p* < 0.05).

**Table 4:** Serum ceruloplasmin levels in WD children carrying R778L mutation

Genotype	Age (years)	Cp (mg/dL)	No. of patients					
			Total	0–5	5–10	10–15	15–20	≥ 20
R778L homozygotes	5.7 ± 3.1	2.3 ± 0.5*	9	9	0	0	0	0
R778L heterozygotes	6.2 ± 3.0	5.2 ± 4.5	97	64	21	6	3	3
No R778L	6.1 ± 2.8	6.1 ± 4.8	207	108	57	29	10	3
Total	6.1 ± 2.9	5.7 ± 4.7	313	181	78	35	13	6

WD, Wilson’s disease; Cp, ceruloplasmin

*Represents statistical significance of serum ceruloplasmin between WD children with and without R778L, *p* value < 0.05

### Diagnostic accuracy of serum ceruloplasmin in Wilson’s disease

**Fig. 2:**
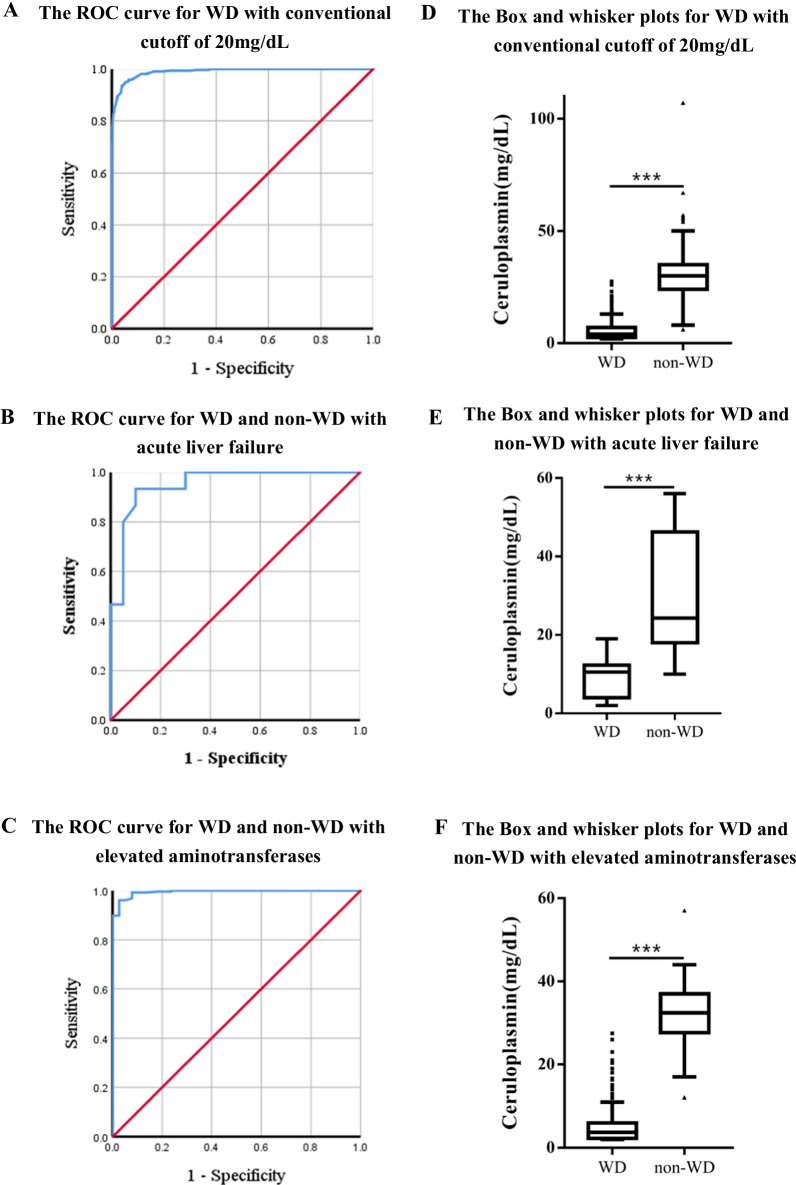
Receiver operating characteristic (ROC) curves of serum ceruloplasmin for the diagnosis of Wilson’ disease (WD). **A** When analyzing WD patients together with heterozygotes and non-WD patients, the area under the curve (AUC) was 0.990 (95% confidence interval (CI), 0.985–0.995). **B** When analyzing WD patients with acute liver failure and non-WD patients with acute liver failure, the area under the curve was 0.952 (95% confidence interval (CI), 0.885–1). **C** When analyzing asymptomatic WD patients and non-WD patients with viral hepatitis, the area under the curve was 0.994 (95% confidence interval (CI), 0.988–1). **D**–**F** Box and whisker plots show the values of serum ceruloplasmin in all WD and non-WD patients (**D**), the patients with acute liver failure and the patients (**E**) with elevated aminotransferases (**F**). The black line represents the consensus cutoff of serum ceruloplasmin (20 mg/dL). The dash lines in **D**–**F** represent the cutoff values obtained in the ROC curves of **A**–**C** (16.9 mg/dL, 16.0 mg/dL and 16.9 mg/dL), respectively.

The ROC curves were constructed by using the data of 317 WD patients, 21 heterozygotes, 372 healthy controls and 154 non-WD patients with renal or hepatic disease. As shown in Fig. 2A, the conventional cutoff value of 20 mg/dL gave a sensitivity of 98.1% and a specificity of 86.5%, with 63 false positives and 6 false negative. The positive and negative predictive values were 80.8% and 98.7%, respectively. The ROC curve analysis indicated that the cutoff value of serum ceruloplasmin level of 16.85 mg/dL provided the highest AUC value of 0.990 (95% confidence interval (CI), 0.985–0.995), with a sensitivity of 95.9% and specificity of 93.6%. The positive and negative predictive values of serum ceruloplasmin level of 16.85 mg/dL were 89.7% and 97.5%, respectively.

To define the accuracy of serum ceruloplasmin levels for the patients with acute liver failure, the ROC curve was constructed by using the data of 15 WD patients and 20 non-WD patients with acute liver failure. The area under the curve was 0.952 (95% confidence interval (CI), 0.885–1). The ROC curve indicated that the cutoff value of serum ceruloplasmin level of 16 mg/dL gave the highest diagnostic accuracy for WD in the patients with acute liver failure (Fig. 2B).

To further define the accuracy of serum ceruloplasmin for diagnosis of WD in the patients with elevated aminotransferases, the ROC curve was constructed by using the data of 256 asymptomatic WD patients with isolated elevated aminotransferases and 38 non-WD patients with viral hepatitis. The area under the curve was 0.994 (95% confidence interval (CI), 0.988–1). The ROC curve suggested that the cutoff value of serum ceruloplasmin level of 16.85 mg/dL gave the highest diagnostic accuracy for WD with elevated aminotransferases (Fig. 2C).

As shown in Fig. 2D-F, the mean level of serum ceruloplasmin of 317 WD patients was 5.7 ± 4.7 mg/dL, significantly lower than that in all of 547 non-WD children (6.0 ± 4.0 mg/dL) (*p* < 0.001). For the patients with acute liver failure, the mean level of serum ceruloplasmin in WD patients (9.3 ± 4.7 mg/dL) was lower than that in non-WD patients (29.7 ± 14.3 mg/dL) (*p* < 0.001). In the situation with elevated aminotransferases, the mean level of serum ceruloplasmin in the 249 asymptomatic WD patients (5.5 ± 4.7 mg/dL) was significantly lower than that in the 38 patients with viral hepatitis (32.5 ± 7.9 mg/dL) (*p* < 0.001).

## Discussion

The present study recruited the largest number of pediatric WD cases with 317 patients and 372 healthy children for study of age and gender specific serum ceruloplasmin levels. Our results were consistent with previous reports, indicating that serum ceruloplasmin is not suitable for newborn screening [7, 16–18]. However, the present study indicated serum ceruloplasmin level may be one of sensitive biomarkers for diagnosis of WD children older than 6 months.

It is interesting to find healthy boys had significantly higher level of serum ceruloplasmin than the girls, so did in asymptomatic WD patients (Fig. 1). Previous study indicated that serum ceruloplasmin has no gender difference except being higher in girls during puberty due to estrogen stimulation [19, 20]. Our study provided very different findings in both healthy children and asymptomatic WD patients based on a large cohort of pediatric WD patients and healthy controls. Gender difference of serum ceruloplasmin should be considered at diagnosis of WD in childhood, and further investigation is needed.

It has been well known that diagnosis of WD is difficult in the asymptomatic children, and in the patients with acute liver failure [3–5]. Serum ceruloplasmin is one of the major diagnostic criteria, however, it has been reported that only 85% of the asymptomatic WD had diagnostic ceruloplasmin levels [21]. With 261 asymptomatic WD children (82.3% of 317 WD patients), this study assessed the diagnostic value of serum ceruloplasmin for WD and presented different finding. Serum ceruloplasmin was generally much lower in WD patients than healthy children, and only 6 asymptomatic WD children of all (6/261, 2.3%) had normal level of serum ceruloplasmin (> 20 mg/dL). Consistent with the reports from other studies from China and South Korea, our findings indicates that decreased level of serum ceruloplasmin is strongly reflecting the abnormality of copper metabolism and the potential diagnosis of WD [6, 12]. Another 13 WD patients in our study had serum ceruloplasmin between 15 and 20 mg/dL, 3 of whom presented with acute liver failure, indicating serum ceruloplasmin is not a sensitive biomarker for WD in the situation of acute liver failure. Some studies from Europe reported that about 30% of European WD patients had serum ceruloplasmin level > 20 mg/dL [22–24]. The racial difference might be the cause for the difference in serum ceruloplasmin levels, and smaller subject number of those studies may also be a reason.

The ROC curve has been used to investigate the optimal cutoff value of serum ceruloplasmin for early diagnosis of WD. It is controversial for optimal cutoff value of serum ceruloplasmin levels for diagnosis of WD, which is from 11.5 mg/dL to 20 mg/dL [9–13, 25]. It has been reported that in the patients with acute liver failure, the conventional cutoff of serum ceruloplasmin level at 20 mg/dL only provided a diagnostic sensitivity of 21% and specificity of 84% for WD [22, 25]. However, most of these studies had some degrees of bias in patient population, with either very few amount of pediatric WD patients or too much more WD patients with neurological symptoms, therefore may not provide an optimal cutoff value in children [3, 12, 25]. In the present study, the ROC curve analyses showed the optimal cutoff value of serum ceruloplasmin is 16.85 mg/dL, with a sensitivity of 95.9% and specificity of 93.6% for diagnosis of WD in children. Using the conventional cutoff value of serum ceruloplasmin 20 mg/dL, the sensitivity is 98.1% and a specificity is 86.5%. Our finding was consistent with that in another Chinese study for WD adult patients, indicating that the threshold of serum ceruloplasmin of 16.8 mg/dL may be more accurate and favor higher specialty for diagnosis of WD in children as well as adults [12].

Genotypic difference of serum ceruloplasmin was also investigated in this study. The genotype of the *ATP7B* gene was associated with the level of serum ceruloplasmin (Table 4). The patients with the genotype of R778L homozygotes had much lower serum ceruloplasmin level (2.3 ± 0.5 mg/dL) than the patients without R778L (6.2 ± 4.8 mg/dL) (*p* < 0.05). Previous study reported that R778L homozygotes are associated with lower level of serum ceruloplasmin and the early onset of WD with hepatic presentation [26]. Other study reported that truncating mutations in the *ATP7B* gene are also associated with very low serum ceruloplasmin level and an early onset of WD [27]. Nicastro et al*.* reported the potential impact of some genotypes on serum levels of ceruloplasmin in WD patients [28] Taken together, genotype in the ATP7B gene is associated with onset of WD and the level of serum ceruloplasmin. Our study further revealed R778L homozygotes may cause decreased serum ceruloplasmin and hepatic damage at very early age.

In conclusion, serum ceruloplasmin level is one of sensitive biomarkers for early diagnosis of WD in children older than 6 months. Gender and genetic difference of serum ceruloplasmin should be considered at diagnosis and treatment for WD patients. The cutoff value of serum ceruloplasmin level at 16.8 mg/dL may provide the highest accuracy for diagnosis of WD.

## Supplementary Information

**Additional file 1**. Supplementary table 1.

## Data Availability

All data generated or analyzed during this study are available from the corresponding author on reasonable request.
