# Supplementary material for: Assessment of the diagnostic value of serum ceruloplasmin for Wilson’s disease in children
Source: BMC Gastroenterol. 2022 Mar 16;22:124. doi: 10.1186/s12876-022-02186-0 (PMC8928661; doi:10.1186/s12876-022-02186-0)
Supplement: Supplementary file 1 — Additional file 1. Supplementary table 1. [file 12876_2022_2186_MOESM1_ESM.docx]

**Supplementary table 1. Clinical and mutational features of 19 WD patients with ceruloplasmin serum levels higher than 15 mg/dl**

| Patients | Sex | Age  (years) | Clinical feature | Cp  (mg/dl) | ALT  (U/L) | AST  (U/L) | Urine Cu  (µg/24h) | *ATP7B* Mutations | |
| --- | --- | --- | --- | --- | --- | --- | --- | --- | --- |
| 1 | F | 10.3 | Acute liver failure | 15.0 | 209.0 | 199.0 | 7629.0 | c.2333G>T | c.2333G>A |
| 2 | M | 8.0 | Asymptomatic | 15.1 | 327.0 | 134.0 | 188.0 | c.2804C>T | c.3809A>G |
| 3 | F | 4.0 | Asymptomatic | 16.0 | 13.0 | / | 76.2 | c.2333G>A | c.2828G>A |
| 4 | F | 4.4 | Asymptomatic | 16.3 | 416.0 | 185.0 | 65.7 | c.2975C>T | c.3007G>A |
| 5 | M | 10.9 | Asymptomatic | 16.7 | 141.0 | 168.0 | 276.6 | c.2128G>A | c.2333G>T |
| 6 | M | 6.7 | Asymptomatic | 18.0 | 88.0 | / | 2149.8 | c.1708-1G>C | c.2975C>T |
| 7 | M | 8.4 | Asymptomatic | 18.0 | 325.0 | 178.0 | 145.9 | c.2755C>G | c.3809A>G |
| 8 | F | 9.2 | Acute liver failure | 18.8 | 232.0 | 241.0 | 1512.5 | c.2333G>T | c.2828G>A |
| 9 | M | 6.3 | Asymptomatic | 18.0 | 297.0 | 113.0 | 156.8 | c.2755C>G | c.2596A>T |
| 10 | F | 4.9 | Asymptomatic | 19.0 | 60.0 | 39.0 | 30.4 | c.2549C>T | c.2621C>T |
| 11 | F | 6.2 | Acute liver failure | 19.0 | 58.0 | 105.0 | 799.4 | c.525dupA | c.2828G>A |
| 12 | M | 5.2 | Asymptomatic | 19.0 | 394.0 | 270.0 | 102.9 | c.3287C>A | c.3646G>A |
| 13 | M | 3.5 | Asymptomatic | 20.4 | 189.0 | 121.0 | 70.5 | c.2804C>T | c.2828G>A |
| 14 | M | 4.3 | Asymptomatic | 21.0 | 167.0 | 92.0 | 60.5 | c.2333G>T | c.2549C>T |
| 15 | M | 3.7 | Asymptomatic | 21.0 | 104.0 | 86.0 | 60.1 | c.2333G>T | c.2549C>T |
| 16 | M | 5.4 | Asymptomatic | 23.0 | 241.0 | 169.0 | 149.1 | c.2333G>T | c.2549C>T |
| 17 | M | 5.5 | Asymptomatic | 26.0 | 75.0 | 129.0 | 155.2 | c.2549C>T | c.3056A>C |
| 18 | M | 3.3 | Asymptomatic | 27.5 | 186.0 | 78.0 | 73.0 | c.2549C>T | c.3884C>T |

ALT, Alanine aminotransferase; AST, Aspartate aminotransferase; Cp, Ceruloplasmin; Cu, Copper.
